# Expanding Horizons in Complement Analysis and Quality Control

**DOI:** 10.3389/fimmu.2021.697313

**Published:** 2021-08-09

**Authors:** Ashley Frazer-Abel, Michael Kirschfink, Zoltán Prohászka

**Affiliations:** ^1^Exsera BioLabs, University of Colorado, Aurora, CO, United States; ^2^Institute of Immunology, University of Heidelberg, Heidelberg, Germany; ^3^Department of Medicine and Hematology, Research Laboratory Semmelweis University, Budapest, Hungary

**Keywords:** complement, laboratory analysis, quality control, diagnostic test, assay performance

## Abstract

Complement not only plays a key role in host microbial defense but also modulates the adaptive immune response through modification of T- and B-cell reactivity. Moreover, a normally functioning complement system participates in hematopoiesis, reproduction, lipid metabolism, and tissue regeneration. Because of its powerful inflammatory potential, multiple regulatory proteins are needed to prevent potential tissue damage. In clinical practice, dysregulation and overactivation of the complement system are major causes of a variety of inflammatory and autoimmune diseases ranging from nephropathies, age-related macular degeneration (AMD), and systemic lupus erythematosus (SLE) to graft rejection, sepsis, and multi-organ failure. The clinical importance is reflected by the recent development of multiple drugs targeting complement with a broad spectrum of indications. The recognition of the role of complement in diverse diseases and the advent of complement therapeutics has increased the number of laboratories and suppliers entering the field. This has highlighted the need for reliable complement testing. The relatively rapid expansion in complement testing has presented challenges for a previously niche field. This is exemplified by the issue of cross-reactivity of complement-directed antibodies and by the challenges of the poor stability of many of the complement analytes. The complex nature of complement testing and increasing clinical demand has been met in the last decade by efforts to improve the standardization among laboratories. Initiated by the *IUIS/ICS Committee for the Standardization and Quality Assessment in Complement Measurements* 14 rounds of external quality assessment since 2010 resulted in improvements in the consistency of testing across participating institutions, while extending the global reach of the efforts to more than 200 laboratories in 30 countries. Worldwide trends of assay availability, usage, and analytical performance are summarized based on the past years’ experiences. Progress in complement analysis has been facilitated by the quality assessment and standardization efforts that now allow complement testing to provide a comprehensive insight into deficiencies and the activation state of the system. This in turn enables clinicians to better define disease severity, evolution, and response to therapy.

## Introduction

The complement system is of substantial relevance for the destruction of invading microorganisms and for immune complex elimination [for review, see (1, 2)]. In addition, complement also modulates the adaptive immune response through modification of T- and B-cell responses using specific receptors on various immune cells. Moreover, a normally functioning complement system participates in hematopoiesis, reproduction, lipid metabolism, and tissue regeneration (2). The critical role of the complement system for host defense is also demonstrated by the multiple complement evasion strategies adopted by pathogens (3). Essential intracellular immune modulatory functions of the complement system have recently been discovered promoting the survival and activation of T lymphocytes (4, 5).

**Figure 1:**
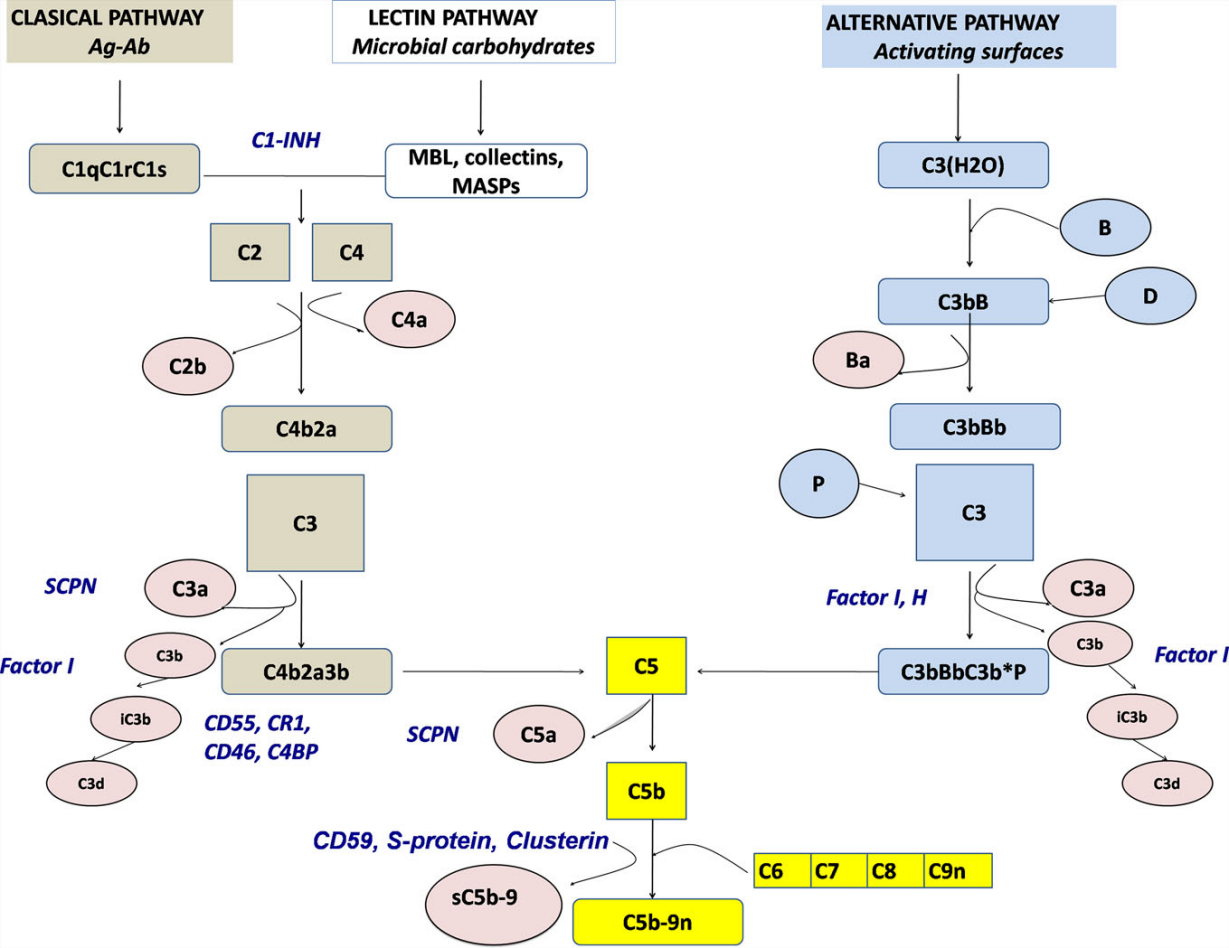
Schematic representation of the complement activation pathways (brown: classical pathway, white: lectin pathway, light blue: alternative pathway, yellow: terminal pathway). Activation products, released into the fluid phase are presented in rose, whereas regulators are presented by blue. SCPN, serum carboxypeptidase N; MBL, mannose-binding lectin.

There are more than 50 complement proteins, including pattern-recognition molecules, proteases interacting in cascade-like fashion, multiple regulatory factors (many of which are cell surface restricted), and receptors (**Figure 1**). Most complement proteins are secreted by the liver and contribute to the acute phase response (6). However, other tissues are also able to produce complement proteins, such as adipocytes for factor D (adipsin), myeloid cells for properdin, and lymphoid cells for a number of components [as reviewed in (7)]. Complement genes are distributed across different chromosomes, with 19 genes comprising three significant complement gene clusters in the human genome (8).

Complement can be activated by any of three main routes: the classical pathway (CP), the alternative pathway (AP), and the lectin pathway (LP) (9). The CP serves as a key effector function of the specific antibody responses, whereas the AP and the LP as ancient parts of the innate immune system are important in first-line antibody-independent defense against bacterial and fungal infections. The terminology of the complement system components refers to the sequence of their discovery, which explains why the cascade is not arranged in a logical numeric order. Components and regulators of the AP are called factors (e.g., Factor B, Factor H) (10).

The CP is activated when the first CP component, C1q, binds to the Fc region of IgG or IgM. In the absence of antibodies, target-bound C-reactive protein (CRP), as well as polyanionic structures on pathogens and apoptotic cells, can also bind to C1q and activate the CP. Upon C1q binding, C1r autoactivates and then activates C1s, which subsequently cleave substrates C4 and C4b-bound C2 to form the C3 convertase (C4b2a) (11). Binding not only of mannose-binding lectin (MBL), a well-known opsonin, and an acute phase reactant with structural similarities to C1q but also of ficolins and collectins to carbohydrate residues on pathogens and altered tissues initiates the lectin pathway (12). Like in the C1 complex, MBL-carbohydrate binding leads to the activation of MASPs (MBL-associated serine proteases), which—like C1s—are able to cleave C4 and C2, thereby connecting the LP to the CP. In contrast to the CP, the AP is activated mainly by non-antibody (non-immunoglobulin) mechanisms. By a mechanism known as tick-over, a permanent low-grade hydrolysis of C3 [C3(H_2_O)] leads—upon binding of factor B and subsequent cleavage by factor D—to the generation of a fluid phase C3-convertase [C3b(H_2_O)Bb], which is stabilized by properdin. In healthy states, this activity is self-limited; however, if newly cleaved C3 binds to pathogens or altered tissue, the AP response is amplified. The regulatory potential of the targeted cells determines if a C3 convertase is formed on the surface and opsonization occurs and the cascade reaction is continued. Once complement is activated by whichever pathway, enzyme complexes (C3 convertases) are generated that cleave C3 into two fragments (C3a and C3b). C3a is the smaller fragment and, like C5a, which is generated later, is a pro-inflammatory signaling molecule (anaphylatoxin). Anaphylatoxins are chemoattractants, they recruit and activate multiple inflammatory cells, including neutrophils and mast cells (13). Receptors for C3b and its metabolic product iC3b on phagocytic cells allow removal of the opsonized targets. Potentially, pathologic immune complexes (containing antibody complexed with viral, bacterial or autoantigens) activate the CP. C3b flags such immune complexes for removal from the circulation by C3b receptor-carrying erythrocytes and selective disposal by phagocytic cells in the reticulo-endothelial system. Cell surface-bound C3b can also trigger the terminal complement cascade. This activation requires factors C5, C6, C7, C8, and multiple C9 to generate the lipophilic membrane attack complex, C5b-9 (MAC), causing target cell death by cell membrane lysis (14).

Because of its powerful inflammatory potential, multiple regulatory proteins are necessary to ensure that potential complement-mediated tissue damage is prevented or at least limited (15). Factor H, Factor I, MCP (CD46), and DAF (CD55), regulating the AP, and C1 inhibitor (C1-INH) and C4b binding protein (C4BP), MCP as well as DAF, controlling the CP and LP, prevent an overactivation of the complement system. C3 convertases are inherently unstable with short half-lives, which helps limit and control complement activation. Excess MAC-mediated complement lysis is prevented by soluble (clusterin, vitronectin) and cell membrane control proteins, CD59 (**Figure 1**). There is increasing evidence that properdin, known as the only positive regulator of the alternative pathway, directly binds to pathogens and apoptotic cells, allowing the generation of C3 convertase on the target surface (16, 17) with subsequent opsonization, i.e. covalent binding of C3b and iC3b.

## Clinical Relevance of Complement

A broad spectrum of clinical disorders is associated either with complement deficiencies or—even more prevalent—with an overactivated and/or dysregulated complement system [for review see (1, 2, 18)].

Complement deficiencies can be either primary (hereditary) or acquired [for review, see (8, 19–22)]. The inheritance is usually autosomal recessive (exception: properdin deficiency: X-linked; Factor B, C1-INH, and MCP/CD46 deficiency: autosomal dominant). Heterozygous carriers usually remain clinically silent. They can be identified through accurate medical history and extended laboratory analysis of the entire family.

From various studies, the prevalence of a congenital complement deficiency has been calculated to be about 0.03%, excluding MBL deficiency, which is estimated to occur in about 5% of the Caucasian population. According to the European Society for Immunodeficiencies (ESID) Registry, deficiencies of complement proteins were responsible for approximately 5% of all primary immunodeficiencies (PID) between 2004 and 2020 (http://esid.org/Working-Parties/Registry/ESID-Database-Statistics; https://cci-reporting.uniklinik-freiburg.de/#/). National registries, however, show a wide variability in the frequencies of these defects, comprising between 1% and up to 30% of all primary immunodeficiencies (23, 24). This may—at least in part—reflect the availability of a comprehensive complement analysis in the respective countries.

The clinical consequences of inherited complement defects fall broadly into three areas: (1) increased susceptibility to infection caused by encapsulated organisms; (2) autoimmunity, in particular systemic lupus erythematosus (SLE); and (3) disorders due to defects of factors controlling, focusing, and limiting complement activation (25).

About 65% of complement-deficient patients suffer from often-recurrent severe invasive infections predominantly caused by encapsulated bacteria (26), whereas viral, fungal, or parasitic infections have only rarely been reported, which is likely because of a compensation of the complement defect by other immune defense mechanisms. Presenting infections due to complement deficiency can include recurrent pyogenic infections (e.g., deep abscess, osteomyelitis, pneumonia), bacteremia, recurrent meningococcal infection, and disseminated gonococcal infection. Neisserial bacteria (meningococcal and gonococcal) are particularly sensitive to complement-mediated attack. However, with the exception of recurrent neisserial infections, patients with recurrent unexplained pyogenic bacterial infections should also be checked for other immune deficiencies, including immunoglobulin or phagocyte deficiency, which are more prevalent than complement deficiency (27).

Complete defects are described for virtually all complement proteins with the exception of serum carboxypeptidase N (SCPN). Secondary deficiencies are caused by inflammation-induced complement consumption, autoantibodies (e.g., against C1q, C1 inhibitor or factor H), decreased synthesis, and/or increased catabolism or protein loss syndromes.

The most frequent complement deficiencies affect C2 and MBL, which often remain clinically silent. The incidence of the hereditary angioedema (Quincke edema) with C1-INH deficiency (HAE-C1-INH) is estimated in 1:10,000 to 1:50,000 (28). Besides controlling complement system activation, C1 inhibitor regulates the fibrinolytic, coagulation, and contact systems. Lack of inhibition results in excessive bradykinin generation, which in turn increases vascular permeability, leading to angioedema. The onset of the disease is early in life, causing attacks of subcutaneous and submucosal edema, which affect the face, periphery, genitals, abdomen, and larynx (29). The upper airway obstruction can result in asphyxia if not treated. An acquired form of C1-INH deficiency mostly occurs before the fourth decade of life and is associated with lymphoproliferative disorders and the presence of autoantibodies to C1 inhibitor. More recently, another type of HAE was identified in patients with normal C1-INH levels. Mutations in genes coding for factor XII (FXII-HAE), plasminogen (PLG-HAE) and, in few families, angiopoietin-1, kininogen-1, or myoferlin have been found in this newly defined group of primary angioedema patients. However, in a significant proportion of HAE patients with normal C1 inhibitor, mutations have not been detected yet (30).

Deficiencies of complement proteins are significantly more frequent in people with specific diseases. In SLE, 30% of the patients have a preexisting complement deficiency (preferentially of C4, C2, and C1) (31), and deficiencies (preferentially of C5–C9 and properdin) are estimated to occur in up to 20% of individuals suffering from disseminated Neisseria infections. With the improvement of PID analysis, in general, and of complement diagnostics, in particular, higher prevalences are expected. In daily practice, some specific clinical presentations (warning signs) raise the possibility of a complement deficiency (21), including meningococcal meningitis > 5 years of age; other recurrent bacterial infections, especially pneumococcus; systemic autoimmune manifestations, especially with onset at a young age and/or familial presentation; angioedema without urticaria; renal and ophthalmic inflammatory disorders.

Clinical consequences of an overactivated and dysregulated complement system include not only immune complex and autoimmune disorders, such as systemic lupus erythematosus (32), various forms of nephropathy, like atypical hemolytic uremic syndrome (aHUS), and C3 glomerulopathy (C3G) (33, 34), ophthalmic disorders, like age-related macular degeneration (AMD) (35), but also organ failure subsequent to ischemia-reperfusion injury (36), sepsis (37), multiple trauma, and burn (38). Furthermore, complement has also been implicated in neurodegenerative disorders, such as Alzheimer’s disease (39), multiple sclerosis (40), and Guillain-Barré syndrome (41). The spectrum of clinical presentations associated with complement dysregulation also includes protein-losing enteropathy (CD55 deficiency) (42) and paroxysmal nocturnal hemoglobinuria (PNH) (CD55+CD59 deficiency) (43).

The inflammatory response due to complement activation induced by artificial surfaces in hemodialysis and extracorporeal circuits may also lead to organ dysfunction. Biomedical polymers differ considerably in their capacity to activate complement (44). Complement activation has been shown to be associated with transient neutropenia, thrombocytopenia, pulmonary vascular leukostasis, and occasionally, anaphylactic shock of variable severity in patients undergoing hemodialysis (45) or cardiopulmonary bypass (46).

Finally, complement activation, if insufficiently regulated, has been reported to enhance tumor progression and to increase metastasis, suggesting its contribution beyond pathogen elimination (47, 48). Complement activation has also been recognized in cancer patients, but its cytotoxic efficacy is often restricted by overexpression of complement surface regulators on the malignant cells (48). There is, however, also evidence that by promoting chronic inflammation, complement activation may support an immunosuppressive tumor microenvironment and activate cancer growth signaling pathways. In line with that, complement activation and reduced expression of membrane complement regulators correlates with poor outcome in cancer patients.

## Complement Testing Addressed in the Standardization Efforts

As with any clinical diagnostics, there is a paramount need for quality, accurate testing. For complement, proper diagnosis involves the determination of the functional capacity and the activation state of the different pathways, the concentration and function of individual components and regulators, the search for complement autoantibodies, as well as the molecular analysis of complement genes (for review see (49–53)). The efforts of the *IUIS/ICS Committee for the Standardization and Quality Assessment in Complement Measurements* (https://iuis.org/committees/qas/subcommittee-for-the-standardization-and-quality-assessment-of-complement-measurements/) have been to evaluate and improve the testing of now 20 different laboratory assessments of complement, all currently focused on the fluid phase complement. The types of complement testing included in these efforts can be broadly grouped into five types as outlined in **Table 1**. These include the following: (1) assessment of the level of the basic components, (2) measurement of the levels and/or functions of the fluid phase control proteins, (3) measures of complement functions, (4) testing for complement directed autoantibodies, and (5) assessment of the fragments and complexes formed during activation. The requirements for that testing have been further influenced by the clinical introduction of complement therapeutics. Although the number of approved drugs that target complement is currently small, there is every indication that this will change soon, as outlined in recent reviews (54, 55). The advent of the complement therapeutics, combined with recognition of the role of complement in a growing number of disorders, has put new demands on the clinical complement laboratory.

**Table 1:** Complement components and potential analytes by pathway.

Section	Classical Pathway	Lectin Pathway	Alternative Pathway	Terminal Pathway
Section 1. Components *Individual Component* *PID: Absent* *Dysregulation: Low* *Activation: Multiple Low* *Inhibition: Normalize*	C1 (C1q, C1r, C1s)	MBL	C3(H_2_O), Properdin	
		Ficolin 1,2,3		
		Collectins		
	C2	C2	Factor B, Factor D	C5
	C4, C3	C4, C3	Factor D	C6, C7, C8, C9
Section 2. Control Proteins PID: Absent* Dysregulation: Absent/Low* *Activation: Low/Unchanged* *Inhibition: Normalize*	C1-INH	C1-INH	Factor H, FHR 1-5, Factor I	
	C4BP + Factor I	MAP-1	Properdin	
Section 3. Function Testing *PID: Absent/Low* *Dysregulation: Low/Uncontrolled* *Activation: Decreased* *Inhibition: Low/Absent*	Liposomal CP			
	CH50 Hemolytic		AH50 Hemolytic	CH50 and AH50 Hemolytic
	ELISA CP	ELISA MP	ELISA AP	ELISA CP, MP, AP
Section 4. Autoantibodies *PID: Normal/Absent* Dysregulation: Present/Absent* *Activation: Unchanged* *Inhibition: Unchanged*	Anti-C1q, Anti-C1s, Anti-CI-INH	Anti-MBL, Anti-Ficolin-3	Anti-FH, Anti-FI, Anti-FB,	
	C4Nef (Anti-C4bC2a)		C3Nef (Anti-C3bBb)	C5Nef (Anti-C3bBbC3b)
Section 5. Activation products *PID: Absent* *Dysregulation: Increased* *Activation: Increased* *Inhibition: Normalize*	C4a, C4b, C2a, C2b, C3a, C3b, iC3b, C3dg	C4a, C4b, C2a, C2b, C3a, C3b, iC3b, C3dg	Bb, Ba, C3a, C3b, iC3b, C3dg	C5a, C5b
				C5a, C5b C5b-9, sC5b-9

The analytes are separated by type. Presented with the type of analyte is the most common outcome of measurements divided into the four broad classes of complement disorders. *Outcome of measurements depends on the actual analyte that is deficient or dysregulated.

PID, Primary Immunodeficiency; CP, Classical pathway; AP, Alternative pathway; LP, Lectin pathway; MP, Microplate.

Still, the most common type of complement testing is the measurement of complement components, most specifically C3, C4, and C1q. The fact that complement factor C3 is present in circulation at levels around 0.1 to 1 mg/ml meant that the tests used originally to look for severe consumption or deficiency had no need to measure in the ng or pg range (56); therefore, most complement component measures utilize the concept of the equivalence zone for efficient measurement. C3, C4, and C1q measurements have also been utilized historically in testing for the rheumatic disease and PIDs (57). With the common and long-standing use of these tests, there are multiple regulatory-approved methods for measuring C3 and C4. For the majority of the other complement components listed in Section 1 of **Table 1**, this is not the case. The benefit of measuring the components is most clear when looking for an individual complement deficiency. When used, as they are in the rheumatic disease, their value often lies in looking for a decrease in the measured C3 and C4 to assess the level and pathway of any ongoing activation of complement leading to consumptions (58). Similar to the measurement of the complement components, the levels of individual fluid phase regulators of complement are also important and utilize many of the same methods. For the measurement of the levels of individual complement components in the context of therapeutic intervention, the measurement of C1-INH levels has the clearest and longest-standing utilization, specifically in the context of hereditary angioedema and C1-INH replacement therapies so it is unsurprising that this area is currently part of specific efforts to improve and standardize (59, 60).

The assessment of the function of the complement system has also been a long-standing type of complement analysis, particularly the assessment of the classical pathway (**Table 1**, Section 3). Historically complement function has been tested by utilizing red blood cells (RBC) as the target of complement lysis (61). A modification that replaces the RBCs with a synthetic liposome that when lysed releases an enzyme that is easily measured on a standard clinical laboratory chemistry analyzer is in wide use in standard clinical laboratories (62). In addition to these lysis-based methods of measuring function, there is a growing number of 96-well style functional assays (63, 64) that have been developed in recent years. These methods move away from using live cells, instead using activators of the individual pathways and then a readout of pathway function that does not require lysis of a liposome or RBC, but instead uses antibodies to detect the formation of the membrane attack complex (C9 neoepitope). Originally developed as semiquantitative screening assay for complement deficiencies, these methods of complement function testing are more approachable for general immunology laboratories and allow for individualized measurement of all three of the activation pathways for the first time (65). Complement function testing has become key in the assessment of utility of complement inhibiting therapeutics, particularly in the treatment of different forms of thrombotic microangiopathies (TMA), including aHUS (66). For these disorders, the functional testing is utilized primarily to determine if the level of inhibition is appropriate to block complement function sufficiently (66). It is important when utilizing complement testing in this way to not only keep in mind how the drug will affect common complement tests [reviewed in (67–69)] but also how the specific type of complement test may affect the result received (67, 70).

Another type of complement testing with a long-standing but expanding footprint is the area of complement autoantibodies. Autoantibodies to C1q have long been recognized as strongly associated with systemic lupus erythematosus [covered in a recent review (71)]. In addition, antibodies to the C3 convertases and to factor H are well recognized as being causative in complement-related kidney diseases (72). The anti-convertase antibodies are known as nephritic factors (C3Nef, C4Nef, and C5Nef, respectively) and have been recognized for their role in kidney diseases, but they have also been seen in other disorders [reviewed in (73)]. Standardization of the autoantibodies is a particular challenge as most forms of tests are methods developed by individual laboratories and rely on scares resources, but there have been successful efforts to standardize these assays as exemplified by advance method agreement and reagent sharing for testing for factor H autoantibodies (74). This work continues with efforts around standardization of the nephritic factors, in particular.

The final type of complement testing included in the IUIS/ICS quality and standardization efforts currently is the measurement of the fluid phase complement activation products in general and the membrane attack complex specifically (**Table 1**, Section 5). The membrane attack complement (C5b-9, MAC), also known as the terminal complement complex (TCC), is produced upon activation of the terminal pathway of complement leading to formation of a complex of C5b, C6, C7, C8, and C9 (1). When inserted in a membrane, this complex can lead to breach of osmotic stability and lysis. Bound to S-protein (Vitronectin), the sC5b-9 complex is held in the fluid phase; it is this circulating form that is becoming a common measure of terminal pathway activation levels (75, 76) to determine the level of activation or inhibition occurring in a patient (77, 78). As such, a measure of terminal pathway activation sC5b-9/sTCC has gained favor as a potential way to assess the likelihood of a patient to respond to therapeutic complement inhibition (68, 79) and then as a measure of the level of appropriate inhibition (80); however, this has yet to be firmly established (81).

Similar to the measurement of the sC5b-9, assessment of the additional activation markers (e.g., C4d, C3a, C3d, C5a, Bb) can inform on the level and location of complement activation across the pathways. In fact, when complement profiles, consisting of functional activities of different pathways, factor and regulator levels, and activation products are determined in parallel, characteristic patterns may be obtained. By measuring complement profiles longitudinally, it is possible to gain an insight into the extent and pathway location of a complement activation or inhibition (68). Such a combination of testing shows a potential avenue for biopsy sparing as seen in the work by the group of Smith et al. (82). Taken into account that (a large) consumption of complement components can impact the potential amount of its cleavage products, it is recommended to use the ratio of the native component to its cleavage product (e.g., C3a/C3).

In addition to the methods that have already become fairly well established in the modern complement laboratory, there are more novel tests being developed that may soon be added to quality and standardization efforts. As a refinement of looking at complement functions, groups have started to look at the function or inhibition of the individual complement convertases (65, 83). Although these methods have a clear benefit to research into understanding the complement system, they also present a clinical potential to look more closely at the therapeutic level of complement inhibition or dysregulation.

Another area of recent advancement is the potential to gain information by multiplexing complement testing. As complement is a cascade of multiple pathways and multiple components, the value of being able to test across the pathways is clear (84). An example of the potential value of this type of approach is presented in the work of Lennart Hammarström of the Karolinska Institute that has pioneered using dried blood spot samples in conjunction with multiplex immunoassays to detect primary complement deficiencies (85). Taking another approach, the group led by Marien I. de Jonge has demonstrated success using mass spectrometry to profile the complement system (84). These early successes are likely only the start of future multiplex testing in the complement laboratory. These new directions and methods not only present great potential for the clinical immunology laboratory but also present yet more challenges around the question of standardization and external quality assessment program for complement testing.

Importantly, conclusive complement analysis depends on correct sampling and subsequent preanalytical handling of the samples (86–88). With the exception of C3 and C4, for which the method of measurement has been designed, so as to gain stability, most of the complement measures will be affected by these factors. The complement function measures will decrease with poor post draw handling, and the activation markers will increase (87, 88). Serum is best suited for functional analysis of the complement pathways and for measuring the concentration of complement components, as well as autoantibodies, whereas the quantification of activation products needs to be performed using EDTA plasma. Chelating divalent cations, such as Ca^2+^ and Mg^2+^, EDTA at concentrations of 10 mM or higher inhibit complement activation from occurring rapidly *ex vivo* (52, 61). Another important measure to prevent *ex vivo* complement activation, serum and EDTA plasma have to be separated from blood cells as rapidly as possible. Subsequently, they need to be subject to immediate analysis or be frozen at −80°C until assayed or shipped to specialized laboratories (http://www.ecomplement.org/european-complement-labs.html) on dry ice.

## Results of Quality Control of Diagnostic Complement Testing

As with all fields of clinical diagnostics, test standardization and documentation, which is supported by internal and external control programs, is of utmost importance (89) for a high quality of complement analysis. The external quality assurance (EQA) program for diagnostic complement laboratories was first established in 2010, with 12 participating laboratories (90). Initially, eight parameters were evaluated (activity of the three pathways, C3, C4, C1q, C1-INH protein, and activity). This number soon went up to 20 parameters, including additional regulators (factor H, factor I), activation products (C3a, C3d, Bb, sC5b-9), and autoantibodies (anti-C1-INH [IgG/IgA/IgM], anti-C1q, C3Nef, anti-FH). Similarly, the number of participating laboratories grew to a total of 35 laboratories in the 2015 EQA round (90) and to more than 200 in 2021. In 2016, members of the Quality Assurance and Standardization of Complement Measurements group hold a 2-day meeting in Budapest where a joint decision to step to the next level with organizational matters was reached. Since 2016, the EQA program has been organized and evaluated by INSTAND (https://www.instand-ev.de/en/), a German non-profit interdisciplinary institute for quality assurance in medical laboratories. Each year, coded samples are sent to registered laboratories. Because there is no target value or reference method available for complement tests, a consensus value of each assay is determined as the mean (with acceptable range of deviation) of the participant’s data, based on predefined schemes by the program directors. If participant numbers in the various method subgroups for a specific assay allow separate analysis (a number higher than or equal to 8), results are evaluated and reported separately.

**Figure 2:**
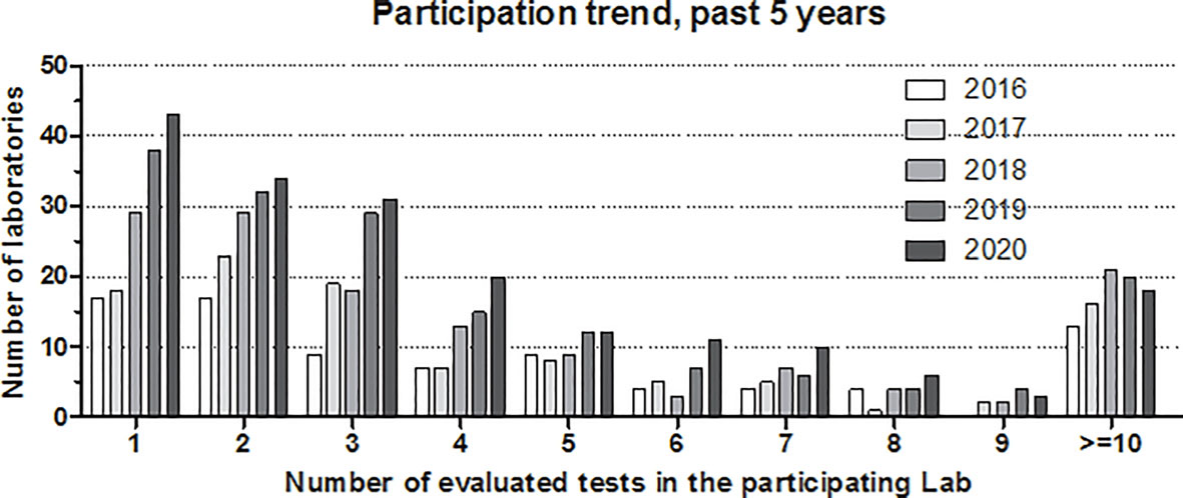
Number of participating laboratories in the external quality assurance program of diagnostic complement laboratories. Participation trends in the past 5 years (2016–2020) are shown separately by the number of tests evaluated in the given laboratory. Note: laboratories participating with more than nine tests are merged as “≥10.”.

**Figure 2** shows the development of participation in the complement EQA program, where results are stratified according to the number of tests evaluated in the given laboratory/year. A clear increase in participation has occurred over the past 5 years, with the highest rise in the number of laboratories evaluating only a few tests (one to four). These laboratories are mainly clinical immunology-oriented, offering complement tests beyond C3 and C4 (e.g., classical pathway activity, C1-INH activity, and anti-C1q). A small increase in the number of laboratories with five to nine tests can also be observed; these are laboratories characteristically offering an extended spectrum of complement tests for either angioedema, glomerulonephritis, or complement deficiency. However, the number of expert complement laboratories offering at least 10 parameters (pathway function multiple autoantibodies, activation products and multiple complement inhibitors) is still limited.

**Figure 3:**
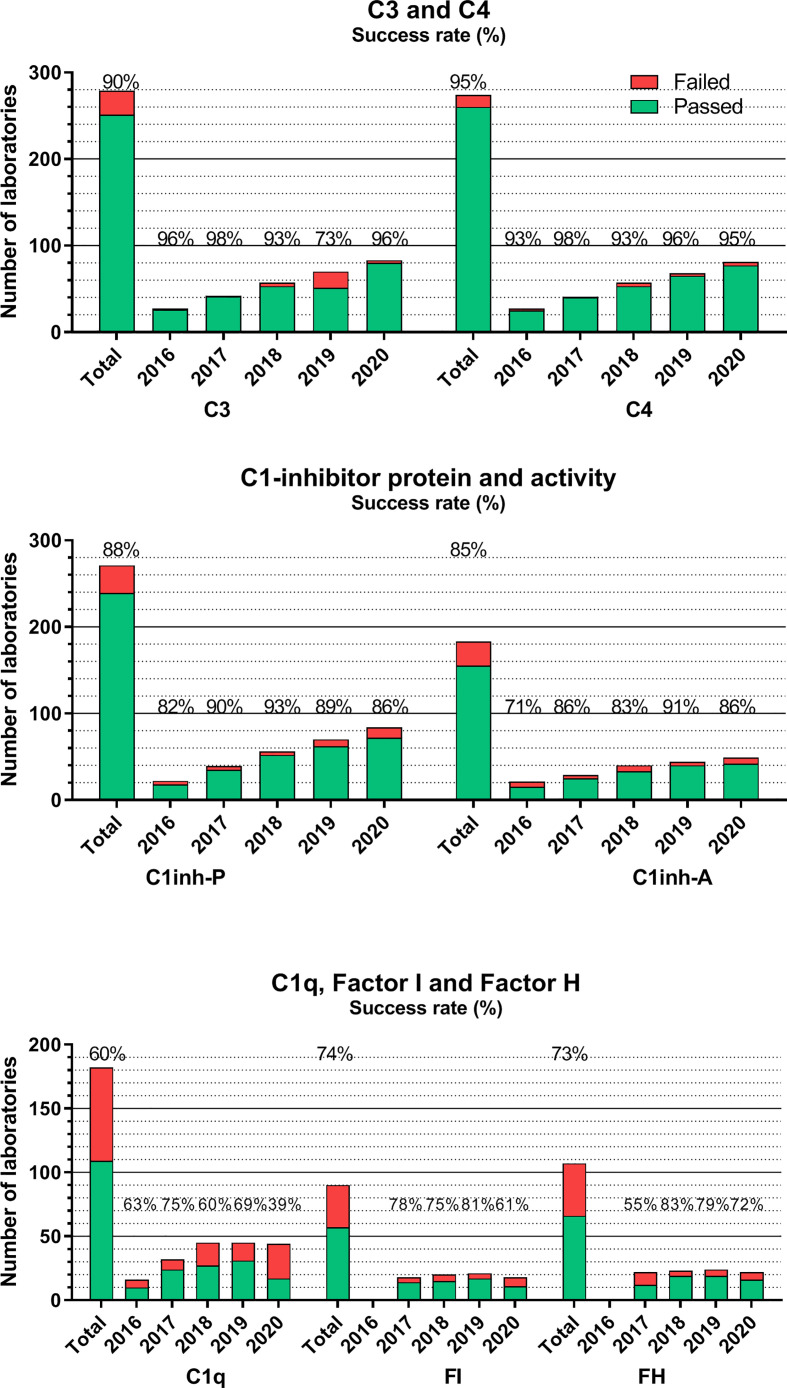
Number of laboratories that “failed” or “passed” in the given year in the EQA program for C3, C4, C1-INH protein, and activity, C1q, factors H and I. Success rate was calculated as frequency of laboratories with ‘passed’ results among all the participants. “Total” indicates the average success rate for the whole group in the past 5 years (2016–2020). Note, that laboratories using commercial nephelometry or radial immunodiffusion (RID) assays have consistently better success rates than laboratories using in-house ELISA or homemade RID. The lack of uniform calibration and a frequent use of ill-defined “units”/ml, both excluded the possibility to evaluate such results in the EQA program (the size of the homogenous method/dimension groups is too low). This is a factor in the increasing proportion of laboratories without certificate.

**Figure 4:**
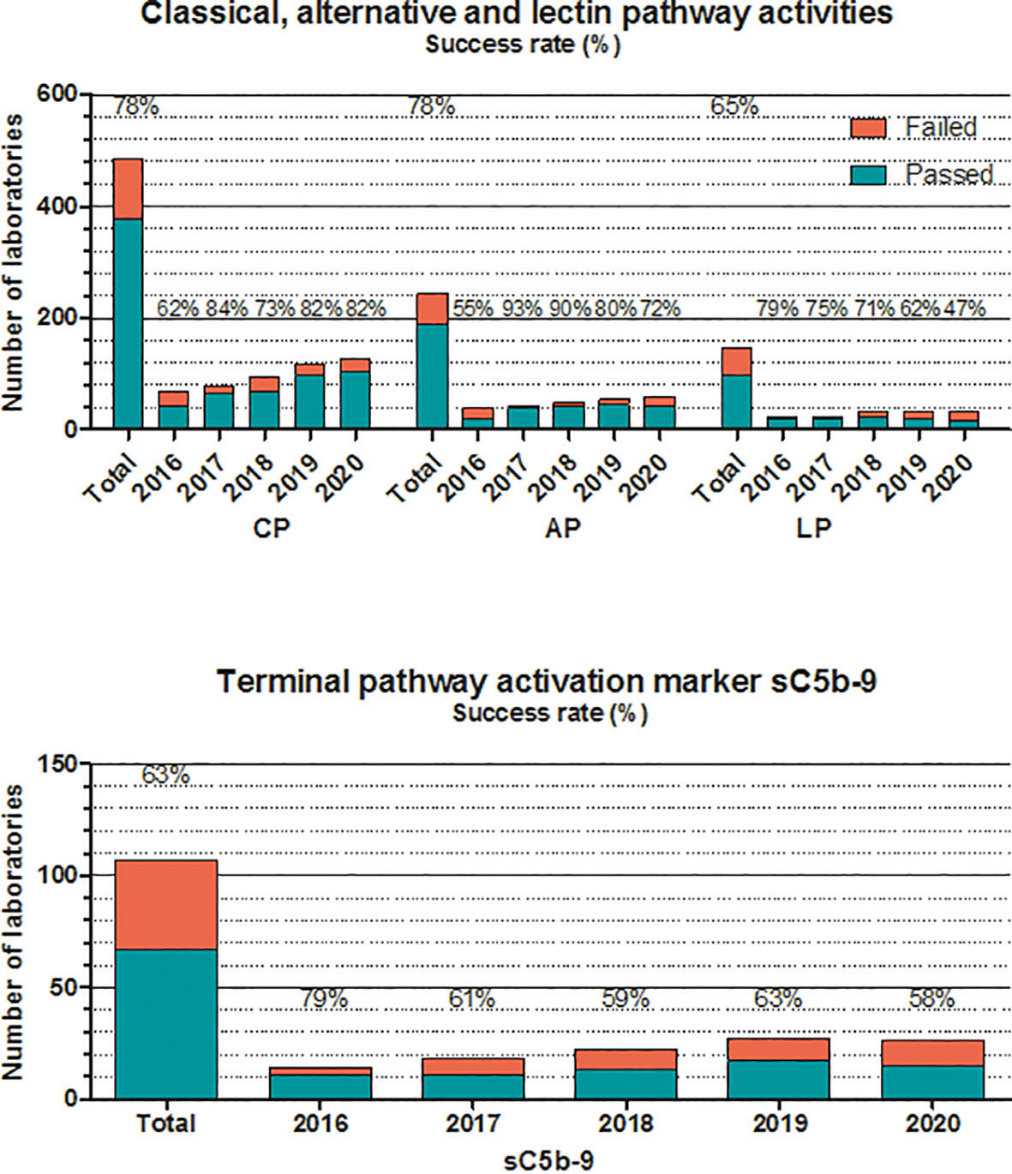
Number of laboratories that “failed” or “passed” in the given year in the EQA program for classical, alternative, or lectin pathways, and terminal pathway activation marker sC5b-9. Success rate was calculated as frequency of laboratories with “passed” results among all of the participants. “Total” indicates the average success rate for the whole group in the past 5 years (2016–2020).

**Figure 5:**
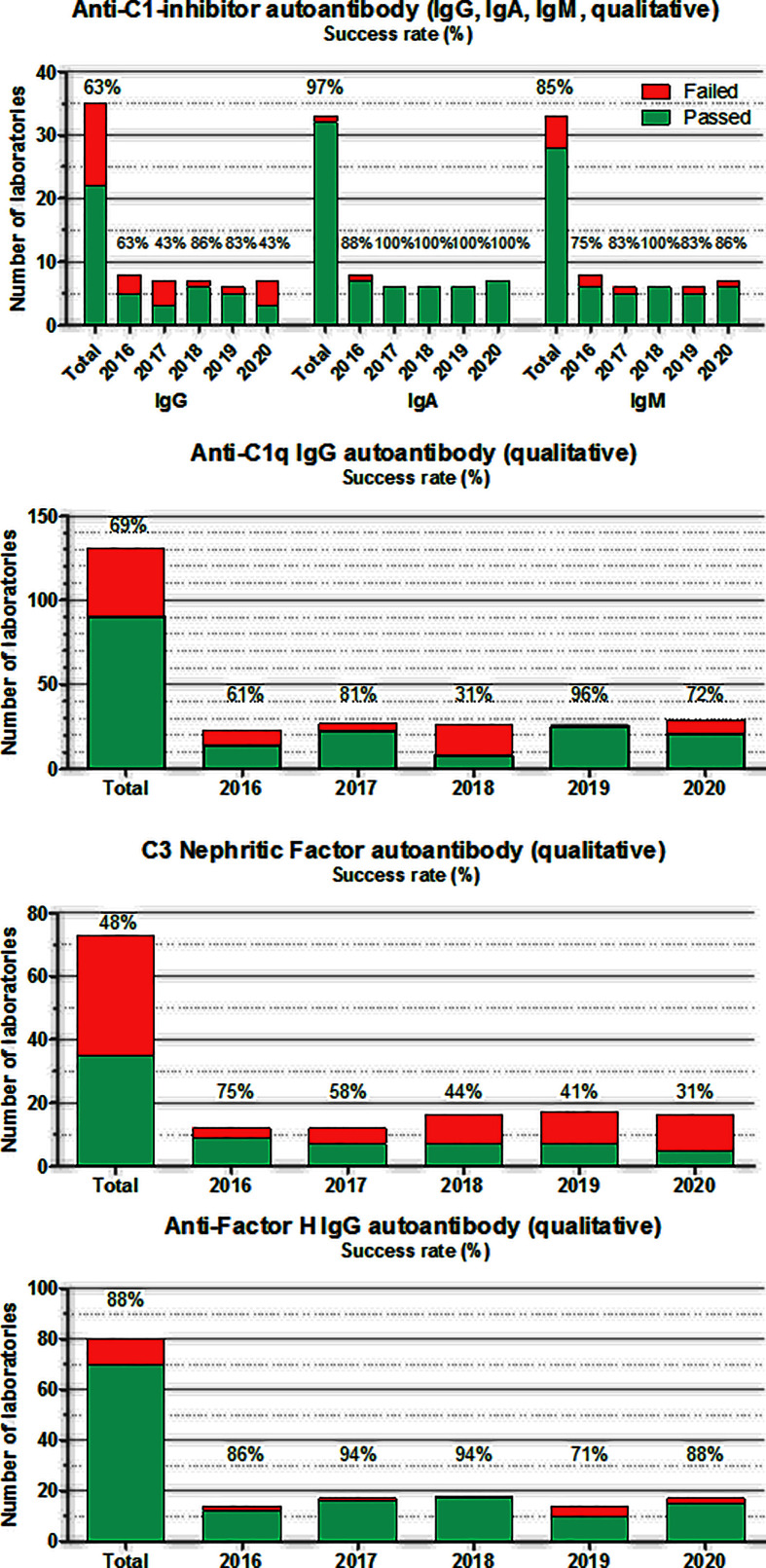
Number of laboratories that “failed” or “passed” in the given year in the EQA program for autoantibodies against C1-INH, C1q, Factor H, and C3 nephritic factor. Success rate was calculated as frequency of laboratories with “passed” results among all of the participants. “Total” indicates the average success rate for the whole group in the past 5 years (2016–2020).

**Figures 3**–**5** show success rates and participant numbers for individual tests in the past 5 years. There are—among others—several sample, method, platform, or calibration-related factors that together determine success rates; the field of diagnostic complement testing is particularly sensitive to several of those factors. The highest success rates (consistently >90% with one exception) were observed for C3 and C4 (**Figure 3**). The two widely used methods (nephelometry and turbidimetry) for C3 and C4 both resulted in equally high performance on all platforms. Similarly, well-performing assays are those for C1-INH protein (88%) and activity (85%). For C1-INH protein, we observed a method-based difference, because the two thirds of the laboratories using nephelometry had consistently better performance (>90%) than those using other methods (mainly ELISA, radial immunodiffusion, or turbidimetry). For C1-INH activity determinations with chromogenic-substrate- or ELISA-based tests yielded an equally high performance. It must be noted that among the 20 assays evaluated in the complement EQA program only C1-INH protein, C3 and C4 are parameters where the majority of the participants use the same method (nephelometry) that is calibrated with international serum protein calibrators regularly used for various serum protein assays on the nephelometers. This appears to be an important determinant of the good analytical performance of these assays.

The situation for additional complement proteins is sharply different, as presented also in **Figure 3**. Average success rates in the past 5 years for C1q, factor H, and factor I never reached 80% in any of the years, without a true increase in the number of participating laboratories. These results are most probably related to multiple factors, such as the frequent use of laboratory-developed assays plus the lack of calibration or agreement on the dimension used to calculate assay results. Measuring the activity of the classical complement pathway (**Figure 4**) provides another illustrative example for this method/dimension problem: the three methods (hemolysis based on sheep red blood cells (SRBC), liposome-based assays and ELISA) with the three widely used dimensions (hemolytic units [CH50/mL], percent lysis of normal serum, and various units/mL) make it sometimes difficult to form appropriate and reasonably sized groups for data evaluation. However, with a higher number of participating laboratories and harmonization of methods over the last 2 years, the performance appears to improve.

For activity measurements of the alternative pathway, the two widely used methods are ELISA and hemolytic assays, whereas for the lectin pathway, ELISA is the only available method (**Figure 4**). Success rates vary between fair and good (50%–93%) in the past 5 years, without a notable trend in the results or differences between the two methods (where available). The same is true for the determination of the terminal pathway activation marker sC5b-9. Results of the ELISA, the only method available, vary between 58% and 79%, despite the fact that 80% of the participants use the same commercial kit for analysis. It should be noted that participants, applying non-commercial assays for sC5b-9, reported consistently poorer results in the past years.

For autoantibodies against complement proteins and inhibitors, the situation is approximately the same in the past 5 years (**Figure 5**). Testing complement autoantibodies is far from being standardized, although some laboratories (especially for anti-FH and anti-C1-INH) attempt to harmonize assay readouts and calibration (74, 91). Despite all efforts, the process of method harmonization and calibration is not yet completed. Therefore, for these analytes, results are evaluated only by qualitative manner reporting readouts compared with their own reference ranges (pos/neg). Anti-C1-INH autoantibodies for the identification of patients with acquired C1-INH deficiency are measured in only a few (5-7) laboratories worldwide. Results of anti-C1-INH have been inconsistent in the past years; therefore, a reference material was developed in the FüstGyörgy Complement Diagnostic Laboratory, Budapest, to calibrate and control the assays. This anti-C1-INH calibrator material is available for all laboratories, participating in this EQA program (please contact the corresponding author of this paper). Anti-C1q analysis is done routinely (mainly by commercial assays) in several immunology and complement laboratories (about 30–40). Here again, results of commercial assays performed better compared with homemade assays. Nephritic factors (92), including C3 nephritic factor against the alternative pathway C3-convertase, are poorly defined functional autoantibodies posing difficulties in laboratory evaluation. There are several different methods (with the SRBC hemolysis-based original method (93) as the current gold standard), which are used in the few laboratories offering this determination as part of the routine workup; the results are largely divergent, even if evaluated qualitatively. There is a clear need for assay development in this area because of the lack of available commercial assay for this autoantibody. Finally, performance of anti-FH autoantibody determination is good, despite the fact that the majority of the laboratories use homemade assays. This achievement is most probably related to the shared protocol and calibrator material offered by the Paris complement Lab (94).

## Conclusions

With the advent of targeted complement therapeutics, several complement-mediated diseases have become manageable; hence, diagnosis, prognosis, and follow-up on treatment efficacy in such diseases become a new task for diagnostic complement laboratories. With the recognition of this unmet need, the initiation and organization of an external quality assurance and standardization program for diagnostic complement laboratories helped to speed up developments in this area. The number of participating laboratories increased in the past years, hence, high-quality, extended complement service is more widely available for the patients and treating physicians. Although the quality improvement is not homogenous for all analytes and assays in the field, the most important measurements show clear progress in complement diagnostics.

## Author Contributions

AF-A, MK, and ZP: Conceptualization, writing of the manuscript, critical reading, and approving of the final version. All authors contributed to the article and approved the submitted version.

## Funding

The research was financed by the National Office for Innovation and Research “Befektetés a jövőbe” (2020-1-1-6-JÖVŐ-2021-00013) to ZP.

## Conflict of Interest

The authors declare that the research was conducted in the absence of any commercial or financial relationships that could be construed as a potential conflict of interest.

## Publisher’s Note

All claims expressed in this article are solely those of the authors and do not necessarily represent those of their affiliated organizations, or those of the publisher, the editors and the reviewers. Any product that may be evaluated in this article, or claim that may be made by its manufacturer, is not guaranteed or endorsed by the publisher.
